# Mechanisms of plant cell wall surveillance in response to pathogens, cell wall-derived ligands and the effect of expansins to infection resistance or susceptibility

**DOI:** 10.3389/fpls.2022.969343

**Published:** 2022-08-23

**Authors:** Delia A. Narváez-Barragán, Omar E. Tovar-Herrera, Arturo Guevara-García, Mario Serrano, Claudia Martinez-Anaya

**Affiliations:** ^1^Instituto de Biotecnología, Cuernavaca, Mexico; ^2^Centro de Ciencias Genómicas, Universidad Nacional Autónoma de México, Cuernavaca, Mexico

**Keywords:** expansin, cell wall oligosaccharides, microbial pathogen, defense response, resistance/susceptibility

## Abstract

Cell wall integrity is tightly regulated and maintained given that non-physiological modification of cell walls could render plants vulnerable to biotic and/or abiotic stresses. Expansins are plant cell wall-modifying proteins active during many developmental and physiological processes, but they can also be produced by bacteria and fungi during interaction with plant hosts. Cell wall alteration brought about by ectopic expression, overexpression, or exogenous addition of expansins from either eukaryote or prokaryote origin can in some instances provide resistance to pathogens, while in other cases plants become more susceptible to infection. In these circumstances altered cell wall mechanical properties might be directly responsible for pathogen resistance or susceptibility outcomes. Simultaneously, through membrane receptors for enzymatically released cell wall fragments or by sensing modified cell wall barrier properties, plants trigger intracellular signaling cascades inducing defense responses and reinforcement of the cell wall, contributing to various infection phenotypes, in which expansins might also be involved. Here, we review the plant immune response activated by cell wall surveillance mechanisms, cell wall fragments identified as responsible for immune responses, and expansin’s roles in resistance and susceptibility of plants to pathogen attack.

## Introduction

Throughout their lifetime plants interact with the microbiota that lives on and inside their tissues, including opportunistic or pathogenic microorganisms, which are controlled by plant physical barriers and multi-layered inducible defense responses (Lemanceau et al., 2017; Choi K. et al., 2021). The first barrier is the cuticle, mostly composed by cutin and epicuticular and intracuticular waxes localized on the aerial parts of the plants (Serrano et al., 2014). The secondary physical barrier is the cell wall, a dynamic and complex structure mainly composed of microfibrils of cellulose in the form of ß-1,4-linked glucan chains, interconnected with xyloglucan and embedded in a hydrated matrix of pectin (Wang et al., 2015; Cosgrove, 2018, 2022). Physicochemical characteristics of the cuticle and the cell wall impact on the composition of the plant microbiome for microbial selection and establishment (Bulgarelli et al., 2013; Aragón et al., 2017; Molina et al., 2021). To interact with their hosts, pathogens require specific activity levels of plant cell wall degrading enzymes such as pectate lyases, cellulases, xylanases, and proteases to digest the cell wall polysaccharides and structural proteins (Bellincampi et al., 2014; Mitsumasu et al., 2015). Digestion fragments play multiple roles in the development and pathogenesis of microbes, but they also trigger plant defense responses and determine the specificity of the plant-pathogen interaction. In particular, short polysaccharide oligomers derived from the cell wall belong to the type of self-recognized molecules known as Damage-Associated Molecular Patterns (DAMPs), and are potent defense-response inducers (Hou et al., 2019; Gigli-Bisceglia et al., 2020), and thus, their application could serve as a cheap and efficient priming treatment to boost plant resistance to prevent infection (Quintana-Rodriguez et al., 2018). For this reason, it is important to explore the characteristics of a range of active glycans such as their optimal activating degree of polymerization, defense-inducing concentration and mode of action, as well as identifying their membrane receptors (Molina et al., 2022). Computational models have for instance been used to determine detailed information of the possible conformation of known interactor pairs [for example receptor: CERK1, with ligand: 1,4-β-d-(GlcNAc)6] with the purpose of predicting and detecting receptors and the signaling pathways leading to resistance (del Hierro et al., 2021). A more challenging aspect of the study of cell wall integrity (CWI) surveillance mechanisms is the perception of modified physical properties and its translation into biochemical signals perceived by membrane receptors (Wolf, 2017). Unexpected changes in cell wall stiffness, surface tension, or barrier properties are in some instances caused by expansin proteins, which are non-enzymatic cell wall remodeling agents (Georgelis et al., 2014; Cosgrove, 2015, 2016). Ectopic expression, constitutive overexpression or exogenous addition of expansins affect plant resistance or susceptibility to infection either by direct modification of the cell wall or by triggering plant immune responses (Marowa et al., 2016; Narváez-Barragán et al., 2020). Here, we review aspects of CWI surveillance mechanisms focusing on stimuli from pathogen colonization, and on cell wall digestion fragments as inducers of defense responses and analyze how these relate to expansin-induced changes in host susceptibility to infection.

## Plant cell wall integrity surveillance

The plant cell wall is synthesized at different levels in a coordinated manner (Cosgrove, 2018). Cell wall polysaccharide content varies according to developmental status (Zablackis et al., 1995), but cellulose, formed at the cell surface by mobile cellulose synthase complexes (CSC), is the most abundant polysaccharide of the cell wall (Grones et al., 2019). In addition, the cell wall contains complex combinations of macromolecules, including hemicelluloses, pectin and lignin, constituting a dynamic structure that can change its composition and properties during plant development without compromising its integrity (Rongpipi et al., 2019). The cell wall is also modified in response to different types of stress (Bacete and Hamann, 2020; Rui and Dinneny, 2020). It is well known that alterations to the CWI activates plant defense responses that in some cases provoke resistance to pathogens (Srivastava et al., 2017; Engelsdorf et al., 2018; Li et al., 2019; Wolf, 2022).

### Osmo- and mechano-sensing

Reduction of cellulose biosynthesis alters CWI inducing cell wall damage that is sensed through diverse mechanisms, including osmo- and mechano-perception, which activate signaling pathways impacting defense responses (Molina et al., 2022). The identity of the components acting upstream of the osmo- and mechano-receptors is practically unknown, and indeed there is little experimental evidence to relate them to responses to pathogens (Bacete and Hamann, 2020). Several microorganisms cause cell wall damage, and it is important to identify cell wall components that alert the cell of microbial invasion. The best characterized osmo-receptors in plants are the *Arabidopsis thaliana* histidine protein kinases (HPKs) (Pham et al., 2012). The *A. thaliana* genome contains eleven HPKs, of which five have been recognized as ethylene receptors, three as cytokinin receptors and the remaining three have no known activity as hormone receptors, although they participate in other signaling processes including responses to biotic and abiotic stresses (Pham et al., 2012; Hofmann et al., 2020). An additional mechanism to cope with physical forces is mediated by mechano-sensitive ion channels (MSIC) and mechano-sensitive like channels (MSL) located at the inner chloroplast envelope (Haswell and Meyerowitz, 2006; Bacete and Hamann, 2020). Based on structural homology with an MSIC originally described in *E. coli*, MSICs have been identified across all kingdoms. In *A. thaliana*, ten homologs exist (MSL for MscS-Like) located in the plasma, inner chloroplast envelope and inner mitochondrial membrane, and have functions related to osmoregulation and two processes clearly related to pathogen responses: cell death and the redox state (Guerringue et al., 2018; Bacete and Hamann, 2020; Schlegel and Haswell, 2020). Functional characterization of plant HPKs and MscS-Like channels is incipient and further research is needed.

### Leucine-rich repeat receptor-like protein kinases

Although diverse response pathways to perceived stimuli exist, most described cell wall-derived elicitor signaling pathways operate through receptor-like kinase (RLKs), which constitute one of the largest gene families in plants, with more than 600 members in *A. thaliana* (Jose et al., 2020). The basic structure of an RLK includes a variable extracellular domain (ECD), a transmembrane region and a cytosolic domain with kinase activity, for phosphorylation of various proteins during downstream signaling (Dievart et al., 2020). The largest subfamily of RLKs, which includes members essential for plant development and immune responses, is one for which the ECD is made up of leucine-rich repeats (LRR-RLK) (Haswell and Meyerowitz, 2006). This class of receptors generally heterodimerize with members of the subfamily SOMATIC EMBRYOGENESIS RECEPTOR KINASEs (SERKs), forming a receptor with two kinase domains that are self and hetero-phosphorylated at multiple residues (Zhou et al., 2019). Once activated, heteromeric complexes phosphorylate other components of the pathway forming a signaling cascade that includes Receptor-Like Cytoplasmic Kinases (RLCKs), Mitogen-Activated Protein Kinases (MAPKs) and/or calcium-dependent protein kinases (CDPKs), ultimately targeting transcription factors to drive gene expression changes in response to a particular ligand (Zhang et al., 2018). Regulation of these pathways includes the activity of pseudokinases and phosphatases, modulation of component abundance and ubiquitination and post-translational modifications, such as glycosylation, acetylation, etc. (Yuan et al., 2021). Thus, the diversity of responses controlled in this manner is enormous, difficult to study and harder to elucidate. Interestingly, other RLK subgroups contain extracellular domains with carbohydrate-binding motifs, being interesting candidates for sensing cell wall-related signals that potentially derive from interactions with pathogenic microorganisms (Zhu et al., 2021). The LRR-RLK family constitutes the largest plant-specific clade of the eukaryotic kinase superfamily and several of its members have essential roles in many aspects of plant development and immunity (Yang et al., 2016; Jose et al., 2020). Despite their importance in defense mechanisms, functional characterization of LRR-RLKs is incomplete, mainly due to high functional redundancy. Even so, some receptors have been extensively studied, generating important information about their role in plant immunity. Such is the case of BRASSINOSTEROID INSENSITIVE1 (BRI1) originally identified as the receptor for plant hormone brassinosteroid and more recently observed as being involved in plant immunity signaling (Lozano-Elena and Caño-Delgado, 2019; Choi J. H. et al., 2021). Heterodimerization of BRI1 with coreceptor BRI1-ASSOCIATED RECEPTOR KINASE1 (BAK1/SERK3) directs the transphosphorylation for Ca^2+^-dependent proteolytic cleavage of BAK1 that is critical for its location at the membrane and its activity to phosphorylate BOTRYTIS-INDUCED KINASE1 (BIK1), an essential control point in plant immunity (Lozano-Elena and Caño-Delgado, 2019; Zhou et al., 2019; Choi J. H. et al., 2021). BAK1 can form a co-receptor complex with MIK2 (LRR-RK MALE DISCOVERER 1-INTERACTING RECEPTOR LIKE KINASE2), reported as the receptor for SERINE RICH ENDOGENOUS PEPTIDE (SCOOP), which is required for resistance to *Fusarium oxysporum*, a root invading fungus causing vascular wilt disease through xylem colonization (Edel-Hermann and Lecomte, 2018). Interestingly, *F. oxysporum* proteome includes SCOOP-like sequences that seem to trigger plant immunity (Van der Does et al., 2017; Hou et al., 2021; Rhodes et al., 2021).

FEI1 and FEI2 are another pair of RLKs involved in CWI signaling; both interact with an arabinogalactan protein (SOS5/FLA4) involved in salinity tolerance and cellulose biosynthesis, as well as with at least two isoforms of AMINOCYCLOPROPANE 1-CARBOXYLIC ACID (ACC) SYNTHASE (ACS), thus relating CWI signaling directly with ethylene and indirectly with other growth regulators (ABA and auxins) (Engelsdorf et al., 2018; Seifert, 2021). Regarding the relationship of FEI proteins with defense responses, *Botrytis cinerea* [a necrotrophic fungus causative of the gray mold disease (Dean et al., 2012)] produces multiple siRNAs that interfere with the immune response, one of which (Bc-siR37) affects *FEI2* transcription. In agreement with this observation, At-FEI2 mutant is hypersensitive to *B. cinerea* infection (Wang et al., 2017). Because of a possible requirement of THE1 for *B. cinerea* resistance, FEI1 and THE1 could operate in the same signaling pathway (Qu et al., 2017). However, these results were obtained with the hypermorphic allele *the1-4*, which could lead to misinterpretation of THE1 in resistance to pathogens (Merz et al., 2017).

### *Catharanthus roseus* receptor-like kinase 1-like

The most studied RLK subfamily in relation to cell wall signaling, frequently associated with attack by pathogens, is the *Catharanthus roseus* Receptor-Like Kinase 1-Like (CrRLK1L) (Franck et al., 2018; Ge et al., 2019; Zhu et al., 2021). In *A. thaliana*, this subfamily consists of 17 members, of which seven have been functionally described (Ge et al., 2019). The CWI CrRLK1L sensor THESEUS1 (THE1), originally identified as a suppressor of the dwarfism phenotype that characterizes the *cesa6prc1-1* mutant affected in cellulose synthase, provokes responses that share similarities to those activated by pathogen infections (Hématy et al., 2007; Merz et al., 2017). Cell signaling through THE1 results in the induction of callose and lignin synthesis and enrichment of homogalacturonan, as a compensatory mechanism for the cell wall weakening in *A. thaliana* (His et al., 2001; Caño-Delgado et al., 2003; Hématy et al., 2007; Bacete et al., 2018; Engelsdorf et al., 2018; Rui and Dinneny, 2020) involving jasmonate, ethylene and salicylic acid, which are hormones often related to plant immunity (Caño-Delgado et al., 2003; Hernández-Blanco et al., 2007; Hématy et al., 2007; Hamann et al., 2009; Shigenaga et al., 2017; Bacete et al., 2018; Engelsdorf et al., 2018). Particularly, in *A. thaliana*, impairment of cellulose synthesis enhance resistance to *Ralstonia solanacearum* [a wilt-causing, xylem invading bacteria (Peeters et al., 2013)], *Plectosphaerella cucumerina* [a root associated necrotrophic fungus (Carlucci et al., 2012)] and *B. cinerea* (Hernández-Blanco et al., 2007; Bacete et al., 2018; Engelsdorf et al., 2018). However, it seems that THE1-mediated defense responses are more related to the monitoring of CWI during plant cell growth and mechanical or hypo-osmotic stress, than to pattern-triggered immunity responses (Hématy et al., 2007; Engelsdorf et al., 2018). Cell wall damage induces oxidative bursts that can be misinterpreted as pathogen attack (Bacete et al., 2018). An ubiquitous family of secreted peptides known as RALFs (rapid alkalinization factors, which block proton channels, increase extracellular pH and stop cell growth) are ligands for different RLKs (Murphy and De Smet, 2014). Peptide RALF34 is perceived by THE1, which affects cell growth in response to cell wall damage (Gonneau et al., 2018). Interestingly, the RALFs represent a point of convergence/divergence between various signaling pathways potentially involved in the immune response (Zhang et al., 2020b). Several of these peptides are ligands of RLKs, being FERONIA (FER) undoubtedly the most widely studied (Ji et al., 2020) due to its involvement in processes such as gametophyte recognition during sexual reproduction, developmental processes, cell expansion, signaling of different hormones, tolerance to abiotic stress, and plant-pathogen interactions (Ji et al., 2020). In the last case, FER acts as a sensor of CWI upon necrotrophic pathogen cell wall digestive enzymes activity. Particularly, defects in the biosynthesis and modifications of pectin are sensed by FER, activating the ROP6 GTPase pathway that controls the formation of the puzzle-shaped pavement cells in *A. thaliana* (De Lorenzo et al., 2019; Lin et al., 2022). FER also has a function as a scaffold protein, recruiting a complex that includes the ELONGATION FACTOR THERMO UNSTABLE RECEPTOR (EFR), FLAGELLIN SENSING2 (FLS2), and BAK1 to initiate immune signaling (Stegmann et al., 2017). This scaffold function of FER depends on its interaction with RALF23. In *A. thaliana* the precursor of RALF23 can be processed by SITE-1 PROTEASE (S1P), preventing complex formation and compromising the immune response (Ji et al., 2020). To regulate immune responses, FER associates in a complex with LORELEI-LIKE GLYCOSYLPHOSPHATIDYLINOSITOLANCHORED PROTEIN1 and 2 (LLG1 and LLG2) and RALF23 (Xiao et al., 2019). *A. thaliana fer* mutants are resistant to *Golovinomyces (syn. Erysiphe) orontii* [fungal pathogen that causes powdery mildew (Braun et al., 2019)], suggesting that FER negatively regulates plant immunity to biotrophic pathogens (Kessler et al., 2010). Plant resistance seems to involve ET- and JA-mediated defense pathways since gene marker *PDF1.2* (a plant defensin gene induced by both phytohormones) is highly expressed in *fer* mutants. However, because *PR1* (a responsive marker gene for salicylic acid) is expressed at normal levels and the ROS burst is suppressed after the pathogen challenge, it has been postulated that powdery mildew resistance is independent of salicylic acid and ROS in *fer* mutants (Kessler et al., 2010). FER-mediated resistance mechanisms cannot be generalized, as *fer* mutants are susceptible to infections by *Hyaloperonospora arabidopsidis*, a biotrophic oomycete that provokes downy mildew (Coates and Beynon, 2010) and *Colletotrichum higginsianum*, a hemibiotrophic fungus causative of anthracnose disease (Kessler et al., 2010; Yan et al., 2018). Interestingly, during infection with *F. oxysporum*, FER is targeted by a RALF mimic secreted by the fungus (F-RALF) (Masachis et al., 2016; Thynne et al., 2017), provoking phosphorylation of H^+^–ADENOSINE TRIPHOSPHATASE 2 (AHA2) and activating a fungal activated mitogen-protein kinase (*Fmk1* -indispensable for fungal pathogenicity), causing an increase of extracellular pH and defense-response inhibition (Segorbe et al., 2017; Nunez-Rodriguez et al., 2020). Similar RALFs are present in 26 species of phytopathogenic fungi, opening the possibility that RALF mimics-mediated inactivation of FER and/or other RLKs is employed by phytopathogens to override plant defense mechanisms (Masachis et al., 2016). Interestingly, in response to *Pseudomonas syringae* [a bacterial phytopathogen (Xin et al., 2018)], FER-induced immune signaling is independent of its kinase activity (Song et al., 2021), also *Meloidogyne incognita* [an endoparasitic nematode of the root vascular cylinder (Tapia-Vázquez et al., 2022) produces RALF mimics (*Mi*RALF1/3) that directly bind the extracellular domain of FER, facilitating parasitism (Zhang et al., 2020a). Additionally, proteins ANX1 and ANX2, which are structurally related to FER, form a receptor-coreceptor complex with others RLKs, namely Buddha’s Paper Seal 1/2 (BUPS1/2), and LLG2/3, which perceives RALF signals to promote ROS production. This complex has already been related to the dynamics of pollen tube growth (Boisson-Dernier et al., 2009; Miyazaki et al., 2009), but ROS, in addition to their well-documented role as a wall-modifying agent, also act as a molecular signal that impacts both cell growth and immune responses. Interestingly ANX1 was re-identified in a genetic screen searching for components controlling plant immunity (Mang et al., 2017; Ge et al., 2019). In summary, FER functions can be associated to a positive or negative regulation of immune responses by binding to RALFs or modulating the assembly and activity of RLKs. Despite the many studies on the matter, much research remains to be done to identify FER interactors and the contribution of each one of them to the host-pathogen interaction.

### Wall-associated kinases

Other types of kinases have been implicated in surveillance of the CWI, such is the case of wall-associated kinases (WAKs) that are distinguished from other receptors by the presence of unique epidermal growth factor (EGF) repeats in the extracellular domain. Up to now they are the only reported receptors with the ability to bind both native cell wall pectin during cell expansion and oligogalacturonides derived from mechanical and pathogen-provoked damage to the cell wall (Kohorn and Kohorn, 2012; Kohorn, 2016). The *A. thaliana* genome encodes five WAKs, 21 WAK-like genes (WAKL), and genes for truncated proteins or with EGF variations (Verica et al., 2003). WAKL genes have also been identified in several angiosperms including wheat, maize, rice, and tomato, and in the first three cases, they are involved in immune responses (Li et al., 2009; Yang et al., 2014; Zuo et al., 2015; Wu et al., 2020). Interestingly, other components of the WAK signaling pathway, the MAPK6 and the transcription factors EDS1 (ENHANCED DISEASE SUSCEPTIBILITY1) and PAD4, (PHYTOALEXIN DEFICIENT4), all of which are involved in the response to pathogens, seem to converge on this pathway (Joglekar et al., 2018; Dongus and Parker, 2021).

### Other proteins associated to cell wall integrity signaling

Cell wall leucine-rich repeat extensins (LRX) are a group of cell wall proteins harboring N-terminal leucine-rich repeats (LRR) predicted to bind ligands, and a highly glycosylated C-terminal extensin domain probably involved in the cross-linking of cell wall components, such as pectins. The presence of LRR and extensin domains place the LRX proteins in a great position to sense cell wall signals and transfer this information to downstream components. Three of the eleven LRX encoded in the *A. thaliana* genome, namely AtLRX 3/4/5, bind peptide RALF23, and interact as a complex with FER causing internalization (Herger et al., 2019). Therefore, LRX represent a link between the cell wall and plasma membrane signaling. The complex AtLRX 3/4/5-RALF23-FER was described in regulation of plant growth and salt stress tolerance but given the important implications of FER on plant immunity (Zhao et al., 2018; Herger et al., 2019, 2020), it is reasonable to think that similar complexes could operate in response to pathogens, although this is pending confirmation. Functional characterization of two *A. thaliana* xyloglucan-deficient mutants *xxt1* and *xxt2*, affected in xylosyltransferase genes, revealed a link between the cell wall and transcriptional control of polar auxin transport mediated by PINFORMEDs (PINs) and AUX1 (Park and Cosgrove, 2012). Furthermore, promotion of xyloglucan-derived hepta- to nona-saccharides cleavage by fungal hemicellulases, regulate auxin-induced growth mediated by expansins (McDougall and Fry, 1990; Shavrukov and Hirai, 2016; Bacete and Hamann, 2020), through at least a cognate receptor (Claverie et al., 2018). However, auxin plays an important role in plant defense signaling, and altered expression of several genes involved in the immune responses, such as FEI1, FEI2, and FER downregulation, or WAK1 upregulation, in the *xxt1* and *xxtp2* mutants (Xiao et al., 2016), suggests that perturbations on xyloglucan content, are involved in the signaling processes.

Because CWI signaling pathway is controlled by the activity of different kinase types, it is valid to assume, for balanced purposes, the need for phosphatase activity. In fact, of the 32 members of the group of impotence rescue (*ipr*) mutants, identified as suppressors of the pollen tube (PT) bursting phenotype that characterizes the double *anx1 anx2* mutant plants (Boisson-Dernier et al., 2015), one of them (*ipr7*) encodes for a Type One Protein Phosphatase (TOPP), named ATUNIS1 (AUN1) that, like its homolog AUN2, is a negative regulator of the CWI maintenance required for the tip growth of the PT (Franck et al., 2018). The possible participation of AUN1/AUN2 and other phosphatases in the CWI signaling pathways outside the tip-growth is an interesting hypothesis to be tested over the next few years.

## The cell wall as source of damage-associated molecular patterns

The cell wall is a source of endogenous elicitors that warn of external danger (Keegstra, 2010; Scheller and Ulvskov, 2010; Mota et al., 2018). When broken down by microbes, polysaccharide fragments are detected by membrane Pattern Recognition Receptors (PRRs), triggering the above-mentioned signaling cascade events for the induction of responses to contain or eliminate the progress of invasion (Saijo et al., 2018). Both Microbe-Associated Molecular Patterns (MAMPs) and/or Damage-Associated Molecular Patterns (DAMPs) induce Pattern-Triggered Immunity (PTI), which can be amplified by feedback loops of endogenous peptides (or phytocytokines) synthesis that also activate PRRs (Gust et al., 2017; Ranf, 2018). PRRs are transmembrane single-pass proteins, with an RLK, or without an intracellular kinase domain and thus known as Receptor-Like Proteins (RLP). These receptors show specificity and high affinity (in the nanomolar range) for their ligands, which could be of protein origin, binding PRRs of the leucine rich repeat category; meanwhile PRRs with LysM motifs recognize N-acetylglucosamine and chitooligosaccharides derived from chitin. Some oligosaccharides are strong elicitors and their size, structure and origin have been reviewed (Zheng et al., 2020). Despite the specificity to various activators, downstream events include pathway crosstalk leading to a general PTI response. In the case of DAMP oligosaccharides, once they interact with their receptors, a fast response occurs resulting in some of the following events: intracellular Ca^2+^ transients, oxidative bursts, NO accumulation, phosphorylation events by kinase signaling cascades (MAPK) and cell wall reinforcement (Sun and Zhang, 2022). Induction of defense-related genes to produce secondary metabolites with antimicrobial activity and enzymes to digest the microbial wall structures (chitinases, ß-1-3 glucanases), and activation of the ethylene, salicylic acid and jasmonate pathways are late outcomes of the response. Several publications have reviewed PTI responses (Ranf, 2017, 2018; Yuan et al., 2021). In the following paragraphs we describe the induction of the plant defense triggered by the major cell wall-derived DAMPs.

### Pectin derivatives: Oligogalacturonides and pectin oligosaccharides

To date, the best-characterized cell wall-derived DAMPs are oligogalacturonides (OGs). OGs are short molecules of α-1,4-linked galacturonosyl residues derived from digested pectin due to microbial activity or by wounding (Ferrari et al., 2013; Claverie et al., 2018; Voxeur et al., 2019). Different response outcomes to OGs depend on: the origin of the microbe involved in the degradation of pectin; plant species; degree of polymerization (DP); and chemical modification of the oligomer (for instance methyl or acetyl esterification). Active OGs are in an egg box conformation that is Ca^2+^-dependent with optimal DP >9, however, protoplasts have shown responses to large pectin fragments, suggesting that the active size is restricted to its capacity for free movement through the cell wall matrix for reaching the plasma membrane (Ferrari et al., 2013). Most work on OGs comes from fungal infection studies and much less is known from bacterial-plant interactions, but a pectate lyase from *Xanthomonas campestris* [a systemic xylem pathogen causing black rot disease (Vorhölter et al., 2012; Lowe-Power et al., 2018)] and a endopolygalacturonase from *Pectobacterium carotovorum* [soft rot disease causal agent (Mansfield et al., 2012)] produce OGs with weak inducing activity at low DP (2–4) but which is optimal at DP >8 (Davis et al., 1984; Norman et al., 1999). In *A. thaliana* and other species, OGs interact with receptor WALL-ASSOCIATED KINASE 1 (WAK1), a representative of WAK receptors containing an EGF-like ectodomain and intracellular Ser/Thr kinase domain, which in turn also binds Glycine Rich Protein 3 (GRP3) through a different binding domain, with a suggested desensitizing activity to control the response to OGs. WAK1 mainly binds non or low-methyl esterified OGs through interaction with five positive residues (arginines and lysines), four of which are located near to the N-terminal at the extracellular portion of the receptor and one further apart, which in combination allow high affinity for the ligand (Decreux et al., 2006). However, the general responses elicited by OGs in a wide range of plant species are accumulation of phytoalexins, callose deposition, production of ROS and NO that lead to resistance to *B. cinerea* in *A. thaliana* (Davis et al., 1986; Bellincampi et al., 2000; Galletti et al., 2008; Rasul et al., 2012; Ferrari et al., 2013; Pontiggia et al., 2020). For instance, Howlader et al. (2020) recently reported that spraying *A. thaliana* with oligosaccharides derived from partial hydrolysis of pectin (POS) confers resistance to *Pseudomonas syringae* pv. tomato DC3000 with an optimal concentration of 25 mg/L 3 days before inoculation. ROS, NO, and the expression of genes PR1, PR2, and PR5 all increased with the treatment.

### Cello-oligosaccharides

Other cell wall-derived DAMPs are cello-oligosaccharides (or cellodextrins). Fewer reports exist for the eliciting capacities of cellulose and hemicellulose, but recent evidence has shown that oligomers from these polymers also trigger defense responses with some differences to those originated from OGs. Different outcomes relative to cellulose derivative burst have been observed in grapevine (Aziz et al., 2007), rice (Yang et al., 2021) and *A. thaliana* (Souza et al., 2017). Grapevine responded to cellodextrins with DP >7, which were strong inducers of cytosolic Ca^2+^ transients and of defensive enzymatic activity [chitinase and ß-1,3 glucanase (Aziz et al., 2007)]. In rice the cello oligosaccharides 3^1^-β-D-cellobiosyl-glucose and 3^1^-β-D-cellotriosyl-glucose are sensed by the CERK1-CEBiP receptor complex, inducing a burst of ROS (Yang et al., 2021). In the case of *A. thaliana*, no oxidative response was induced after cellobiose (or higher DP cellodextrins) treatment, but phosphorylation of MPK3 and MPK6 rapidly occurred (with a peak at 10 min), resulting in the induction of *WRKY30* transcription factor and calcium transients. Cellobiose treatment also upregulates genes associated with phytohormone signaling: *SAG101*, *PAD4* for SA; *ACS7* for ET; and *LOX3* and *LOX4* for jasmonate but the treatment failed to deposit callose or suberin to reinforce the cell wall. Although perception of cellodextrins can saturate, the responsible PRR remains unknown, and analysis of the receptor involved in the response to cellulose indicates that THESEUS is not required in the cellobiose pathway (Aziz et al., 2007; Hématy et al., 2007). Compared to hepta cellodextrin in grapevine, cellobiose response induction in *A. thaliana* is weaker, but synergizes the response to chitin, flg22 and OGs when applied simultaneously, suggesting different signaling pathways and an auxiliary response to other danger signals (Souza et al., 2017). Additionally, oxidized and native cellulose- or cello-oligosaccharides (DP from 2 to 10), and C1- or C4-oxidized products oxidatively cleaved from cellulose by the microbial enzymes lytic polysaccharide monooxygenases (LPMOs), activate immunity in *A. thaliana* and tomato and induce resistance to *B. cinerea*. Upon treatment, two receptors, STRESS INDUCED FACTOR 2 and 4 (SIF2, SIF4), are induced and might form a complex with BAK1 and THE1 that detect these molecules (Zarattini et al., 2021).

### Oligoxyloglucans

Xyloglucan is the main hemicellulose of dicot plants, and in a report by Claverie et al. (2018) authors demonstrated that DP 7 heptamaloxyloglucan (and in minor concentrations also higher DPs) obtained from apple pomace is an elicitor (through a still unknown membrane receptor) in grapevine and in *A. thaliana* (Claverie et al., 2018). This oligoxyloglucan induces a fast dose-dependent phosphorylation of MAPKs within the first 10 min of treatment, but without a detectable oxidative burst. Late responses to the oligoxyloglucan include defense-related gene induction of the SA- and camalexin-pathways in *A. thaliana* and *PAL* (phenyl ammonia lyase) and *STS* (stilbene synthase) genes of the resveratrol biosynthesis pathway in grapevine (Claverie et al., 2018). *A. thaliana* mutants of the camalexin-, SA-, ET-, and JA-pathways confirmed their involvement to the presence of oligoxyloglucan as they failed to protect toward a challenge with *B. cinerea* (Claverie et al., 2018). Additionally, cell wall reinforcement with callose deposition is part of the responses induced by oligoxyloglucan (Claverie et al., 2018; Héloir et al., 2019). In the case of arabinoxylan, it was reported that pentose oligosaccharides with arabinose decorations as the pentasaccharide 3^3^-α-L-arabinofuranosyl-xylotetraose (XA3XX) triggered Ca^2+^ transients, ROS burst, and induce MAPK3 and MAPK6 phosphorylation in *A. thaliana* (Mélida et al., 2020). Chemically synthesized mixed-linked glucans or MLGs [β-1,3/1,4-glucans; (1,3;1,4)-β-d-glucans] also trigger immunity, with the trisaccharide MLG43 [β-D-cellobiosyl-(1,3)-β-D-glucose] being the smallest of these molecules to act as a potent pathogen resistance inducer (Rebaque et al., 2021). Other species might also express receptors for MLG as MLG43 protected against oomycete, bacterial and fungal pathogens in *A. thaliana*, tomato, and pepper, which in this case would be detected as foreign molecule because MLG43 is absent in their cell walls, but present in the cell wall of some microorganisms (Barghahn et al., 2021).

Hyperinduction of immune responses activated by cell wall oligosaccharides is thought to be prevented by oligosaccharide oxidation of the (cellodextrins and oligogalacturonides) by FAD-dependent Berberine Bridge Enzyme-like (BBE-like) proteins with 27 members in *A. thaliana*, which render them inactive in this state. Oxidizing oligosaccharides also confer resistance to microorganisms that are unable to metabolize them (Benedetti et al., 2018; Locci et al., 2019). However, Zarattini et al. (2021) showed that C1-oxidized cellobionic acid is still able to induce high expression of defense genes.

## Expansins in susceptibility to infection

Because CWI is key for its barrier functions, modification by cell wall proteins can have distinct outcomes for pathogen susceptibility or resistance. Expansin’s importance in plant-microbe interactions is evidenced by a growing number of reports of expansin involvement in different phenotypes (Cosgrove, 2015). Expansins are a family of plant and microbial proteins with cell wall remodeling roles through their binding capacity to cellulose and other cell wall polysaccharides, and hypothetical non-catalytic disruptive activity of weak bonding between polysaccharides (McQueen-Mason and Cosgrove, 1994; Cosgrove, 2015, 2017). Different subclasses of plant expansins act in many physiological and developmental processes that involve controlled cell wall remodeling, and in particular their contribution in cell wall extension is well known (Cosgrove, 2016), yet expansins are also induced under several stressing conditions (Feng et al., 2019; Kong et al., 2019). Additionally, due to their prevalent induction observed in expression profiling analysis under stress, proteins classified as expansin-like type α and type β (subfamilies EXLA and EXLB), have been associated to cell wall modification resulting in (mainly abiotic) stress tolerance (Abuqamar et al., 2013; Marowa et al., 2016; Guimaraes et al., 2017; Chen et al., 2019; Muthusamy et al., 2020; Zhang et al., 2021; Morales-Quintana et al., 2022). Subfamily X (EXLX), includes microbial expansins, from bacteria, fungi and oomycetes, with structural similarity to ß-expansins (Kende et al., 2004; Nikolaidis et al., 2014). Very few reports on microbial expansin activity on the plant cell wall exist, probably in part due to the difficulty on quantifying their effect, although it is known that they also bind cellulose and plant cell walls, but show only weak extension activity in comparison to plant expansins (Kerff et al., 2008; Georgelis et al., 2014, 2015; Olarte-Lozano et al., 2014). As mentioned before, signs of cell wall modification due to microbial activity or induced by abiotic stress are detected by membrane receptors alerting the cell to mount defense and stress responses, sometimes simultaneously. For instance, ectopic overexpression of EXLB8 from *Arachis* in tobacco results in tolerance to water deficit and resistance to the white mold causative necrotrophic fungus [*Sclerotinia sclerotiorum* (Hegedus and Rimmer, 2005)], and to the nematode *M. incognita*, and to drought and simultaneous nematode infection (Brasileiro et al., 2021). Different cell wall nanomechanical properties (possibly stiffer, but more deformable leaves), brought about by EXLB8 overexpression, and consequently, possibly by other six endogenous EXL genes, was suggested as responsible of the phenotype hampering a successful infection. Various biotic and abiotic stress genes of the jasmonate, ethylene and abscisic acid pathways were also induced, activating a more general and unspecific priming of the host against pathogens with important lifestyle differences (Brasileiro et al., 2021). Contrarily, *A. thaliana EXLA2* down-regulation provides resistance to *B. cinerea* and oxidative stress, although roots become sensitive to salt and cold when the medium contains ABA (Abuqamar et al., 2013). Susceptibility to pathogens might be a combination of a stiffer or more relaxed cell wall directly affecting colonization and the defense responses resulting from these changes, although to this day the trigger remains unknown. A clue as to the chemical nature of a possible ligand is exemplified by treatment of celery petioles and *A. thaliana* leaves with expansin Exl1 from *Pectobacterium brasiliense* strain BF45, which show resistance to a challenge of BF45 and *B. cinerea* 24 h after Exl1 infiltration (Narváez-Barragán et al., 2020). Because Exl1 binds to celery cell corners of cells surrounding xylem vessels, which is a site abundant in pectin and due to the seeming solubilization of a phenolic-substituted polysaccharide from isolated Swiss chard vascular bundles *in vitro*, it has been suggested that pectin could be the ligand triggering ROS bursts, and inducing genes of the jasmonate, ethylene and salicylic acid pathways (Tabuchi et al., 2011; Wang et al., 2016; Tovar-Herrera et al., 2018). More recently, Pi et al. (2022) identified a G-type lectin receptor-like kinase (ERK1, for *expansin regulating kinase*) that responds to the presence of expansin EXLX1 from *Phytophthora capsici* [one of the most harmful hemibiotrophic oomycetes (Pi et al., 2022)] in tobacco leaves, providing resistance to the infection. Although the ligand for the receptor remains to be found, this is a first step toward our understanding of the mechanisms underlying perception of wall modification by expansins.

## Perspectives

**FIGURE 1:**
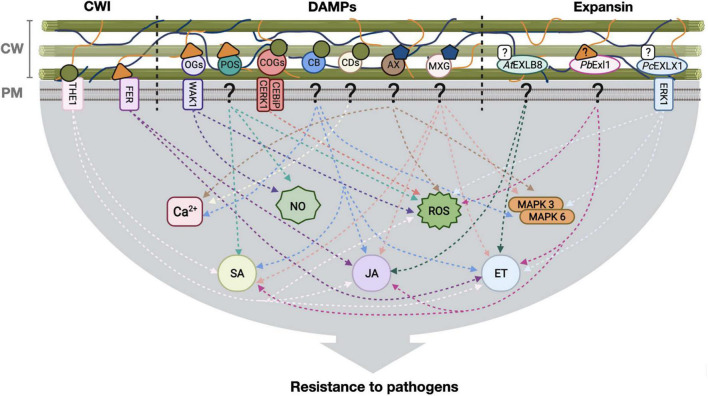
Defense responses activated by plant cell wall modification for pathogen resistance. Cell wall integrity (CWI) surveillance receptors, DAMPs and expansins interact with plant cell wall components and elicit defense responses leading, in some cases, to pathogen resistance. Stimuli from the cell wall is transduced to the cell interior through different pathways (arrows), for the induction of hormone-dependent signaling, NO, ROS and Ca2 + transients. CW, cell wall; PM, plasma membrane, ?: unknown receptor; OGs, oligosaccharides; POS, pectin oligosaccharides; COGs, cello-oligosaccharides; CB, cellobiose; CDs, cellodextrins; AX, arabinoxylan; MXG, heptamaloxyloglucan; *At*EXLB8, *Arabidopsis thaliana* expansin-like B; *Pb*Exl1, *Pectobacterium brasiliense* expansin-like 1 protein; *Pc*EXL1, *Phytophthora capsici* expansin-like 1 protein; THE1, THESEUS1 receptor; FER, FERONIA receptor; WAK1, WALL ASSOCIATED KINASE 1; CERK1-CEBiP, chitin-elicitor receptor kinase 1-chitin-elicitor binding protein receptor complex; ERK1, expansin-regulating kinase 1; NO, Nitric oxide; ROS, reactive oxygen species; MAPK, mitogen-activated protein kinases; SA, salicylic acid; JA, jasmonic acid; ET, ethylene. 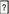 Unknown ligand, ^
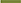
^ Cellulose, ^
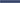
^ Hemicellulose, ^
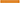
^ Pectin, ^
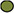
^ Cello-oligosaccharides, ^
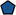
^ Hemicellulose derivatives, ^
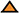
^ Pectin derivatives. Figure created with BioRender.com.

Cell wall modification as a response to abiotic stress or due to pathogen activities induces defense responses resulting in priming of the host locally and systemically, in some cases involving common or shared pathways (Figure 1). Several studies have explored the importance of expansin proteins in this process, being of particular interest those that provide resistance to pathogens, opening the possibility for expansin treatment as a biotechnological application for crop improvement. However, given that a positive effect is not a general outcome, further investigation will tell which expansin treatment would modify the cell wall properties and subsequent defense responses in a manner that is favorable for the host. For this purpose, we need to integrate our knowledge on expansin activity (from both plant and microbial origins) with the mechanisms of the cell wall surveillance and responses to stress. Analysis of possible cell wall physical modification and changes in mechanical properties brought about by expansins, can be done combining traditional techniques with novel methods of cell wall imaging using fluorescence-based probes for different components recently developed (Michels et al., 2020; DeVree et al., 2021). This would shed light on whether resistance or susceptibility are a consequence of a direct cell wall alteration and how this affects microbial invasion, and it will tell whether some expansin types are better than others in providing resistance. With respect to microbial expansins many questions remain unanswered, for instance, which expansin treatment provides resistance (or susceptibility) and to which hosts. Most reverse genetics experiments of bacterial expansins indicate a requirement for proper colonization, except that of Exlx2 from *C. michiganensis* (Tancos et al., 2018), suggesting that similarly to plant expansins, microbial expansin treatment might result in different phenotypes, and thus, each case must be studied independently. Another important question is determining the identity of trigger stimuli that activate defenses after expansin acts on the cell wall. And finally, what pair of receptor-ligand (a detached polysaccharide fragment maybe) is responsible for intracellular signaling? Further research on this topic will be required to explore the biotechnological potential of expansins as possible biocontrol agents.

## Author contributions

All authors listed have made a substantial, direct, and intellectual contribution to the work, and approved it for publication.
